# Genetic Evidence of Serum Phosphate-Independent Functions of FGF-23 on Bone

**DOI:** 10.1371/journal.pgen.1000154

**Published:** 2008-08-08

**Authors:** Despina Sitara, Somi Kim, Mohammed S. Razzaque, Clemens Bergwitz, Takashi Taguchi, Christiane Schüler, Reinhold G. Erben, Beate Lanske

**Affiliations:** 1 Department of Developmental Biology, Harvard School of Dental Medicine, Boston, Massachusetts, United States of America; 2 Endocrine Unit, Massachusetts General Hospital and Harvard Medical School, Boston, Massachusetts, United States of America; 3 Department of Pathology, Nagasaki University School of Biomedical Sciences, Nagasaki, Japan; 4 Department of Natural Sciences, University of Veterinary Medicine, Vienna, Austria; The Jackson Laboratory, United States of America

## Abstract

Maintenance of physiologic phosphate balance is of crucial biological importance, as it is fundamental to cellular function, energy metabolism, and skeletal mineralization. Fibroblast growth factor-23 (FGF-23) is a master regulator of phosphate homeostasis, but the molecular mechanism of such regulation is not yet completely understood. Targeted disruption of the *Fgf-23* gene in mice (*Fgf-23^−/−^*) elicits hyperphosphatemia, and an increase in renal sodium/phosphate co-transporter 2a (NaPi2a) protein abundance. To elucidate the pathophysiological role of augmented renal proximal tubular expression of NaPi2a in *Fgf-23^−/−^* mice and to examine serum phosphate–independent functions of Fgf23 in bone, we generated a new mouse line deficient in both *Fgf-23* and *NaPi2a* genes, and determined the effect of genomic ablation of *NaPi2a* from *Fgf-23^−/−^* mice on phosphate homeostasis and skeletal mineralization. *Fgf-23^−/−^/NaPi2a^−/−^* double mutant mice are viable and exhibit normal physical activities when compared to *Fgf-23^−/−^* animals. Biochemical analyses show that ablation of *NaPi2a* from *Fgf-23^−/−^* mice reversed hyperphosphatemia to hypophosphatemia by 6 weeks of age. Surprisingly, despite the complete reversal of serum phosphate levels in *Fgf-23^−/−^/NaPi2a^−/−^*, their skeletal phenotype still resembles the one of *Fgf23^−/−^* animals. The results of this study provide the first genetic evidence of an *in vivo* pathologic role of NaPi2a in regulating abnormal phosphate homeostasis in *Fgf-23^−/−^* mice by deletion of both NaPi2a and Fgf-23 genes in the same animal. The persistence of the skeletal anomalies in double mutants suggests that Fgf-23 affects bone mineralization independently of systemic phosphate homeostasis. Finally, our data support (1) that regulation of phosphate homeostasis is a systemic effect of Fgf-23, while (2) skeletal mineralization and chondrocyte differentiation appear to be effects of Fgf-23 that are independent of phosphate homeostasis.

## Author Summary

Regulation of phosphate homeostasis is a tightly controlled hormonal process involving the intestine, kidneys, and bone, and imbalance of this homeostasis may influence overall mineralization. Fibroblast growth factor-23 (FGF-23) is a circulating hormone produced in the bone that mainly targets the kidneys to control the activity of the sodium/phosphate co-transporters NaPi2a and NaPi2c. These transporters are responsible for actively reabsorbing phosphate ions into the body to maintain physiological serum phosphate levels. Changes in FGF-23 activity lead to human disorders associated with either phosphate wasting or retention. Genetically altered mice in which Fgf-23 activity is lost exhibit severe hyperphosphatemia accompanied by increased NaPi2a activity, and they develop abnormal bone mineralization. Here we describe a new mouse model in which we eliminated NaPi2a from Fgf-23 null mice and show reversal of hyperphosphatemia to hypophosphatemia, suggesting that NaPi2a is the major regulator of phosphate homeostasis. However, the skeletal mineralization defect observed in mice lacking Fgf-23 function remained unchanged in the absence of NaPi2a in these mice. Thus our data indicate that Fgf-23 has a role in controlling bone mineralization independent of systemic phosphate levels.

## Introduction

Maintaining physiological phosphate balance is essential, not only for skeletal mineralization but also for various important biological activities that include cellular signaling, and biochemical reactions [1]. Acute hypophosphatemia can cause myopathy, cardiac dysfunction, and hematological abnormalities, whereas chronic hypophosphatemia impairs bone mineralization, resulting in rickets and osteomalacia [2]. On the contrary, hyperphosphatemia is associated with vascular and soft tissue calcifications [3]. Understanding the molecular regulation of phosphate homeostasis has, therefore, enormous clinical and biological significance.

The kidney is the major site of hormonal-dependent regulation of phosphate homeostasis, controlling urinary phosphate excretion according to the needs of the body [1]. Phosphate transport across the renal proximal tubular epithelial cells is a sodium-dependent process, driven by the gradient between extracellular and intracellular sodium concentrations, and such gradient is known to be maintained by the basolateral membrane–associated Na^+^/K^+^-ATPase [4].

The identification of distinct phosphate (Pi) transporters has increased our understanding of the mechanisms and regulation of renal and intestinal phosphate handling. The type II family of Na/Pi co-transporters consists of three highly homologous isoforms: type IIa (NaPi2a) and type IIc (NaPi2c) are almost exclusively expressed in the brush-border membrane of the renal proximal tubules [5]–[7], whereas type IIb (NaPi2b) is expressed in the epithelial cells of the small intestine, and is thought to be involved in intestinal phosphate absorption. The NaPi2b co-transporter is not expressed in the kidney [8]. Since renal phosphate transport through NaPi2a is an important mechanism of maintaining phosphate balance, the molecules that directly or indirectly affect NaPi2a can influence phosphate homeostasis.

The critical role of NaPi2a co-transporters in the maintenance of Pi homeostasis was demonstrated by genetic ablation of the murine *NaPi2a* gene by homologous recombination. Mice ablated for the *NaPi2a* gene (*NaPi2a* ^−/−^) exhibit increased urinary phosphate excretion, resulting in hypophosphatemia [9]. Despite comparable serum levels of calcium, phosphate, PTH, and 1,25(OH)_2_D_3_ *NaPi2a^−/−^* mice exhibit a ricketic bone phenotype at 3 weeks of age; these mutant mice show a normal skeletal phenotype comparable to wild-type animals at 6 weeks, and have increased trabecular bone volume at 12 weeks of age [9].

FGF-23 has been shown to be an important regulator of renal Pi handling. FGF-23 inhibits renal phosphate reabsorption by suppressing the expression of NaPi2a and NaPi2c co-transporters [10]. We have recently generated *fibroblast growth factor-23* null (*Fgf-23* ^−*/*−^) mice which are characterized by severe hyperphosphatemia, and increased renal expression of NaPi2a [11],[12]. In view of the fact that FGF-23 is a major regulator of phosphate homeostasis [13]–[16], this study was designed to assess the pathophysiological significance of increased renal expression of NaPi2a in *Fgf-23* ^−/−^ mice. To test such significance, we have established a new mouse model by genetically ablating both *Fgf-23* and *NaPi2a* genes in the same animal, in order to determine whether altered phosphate homeostasis in *Fgf-23* ^−/−^ mice is a *NaPi2a*-mediated process. In addition, using this model, we sought to examine phosphate-independent effects of Fgf-23 on skeletogenesis.

## Materials and Methods

### Animals

Heterozygous- *Fgf-23* and *NaPi2a* mice were interbred to attain wild-type, *Fgf-23^−/−^*, *NaPi2a^−/−^*, and *Fgf-23^−/−^/NaPi2a^−/−^* animals at 3 and 6 weeks. Routine PCR was used to identify the genotypes of various mice as described previously [9],[11]. All studies performed were approved by the institutional animal care and use committee at the Harvard Medical School.

### Bone Densitometry

Bone mineral density (BMD) and bone mineral content (BMC), were determined on 3- and 6-week-old wild-type, *Fgf-23^−/−^*, *NaPi2a^−/−^* and *Fgf-23^−/−^/NaPi2a^−/−^* mice using the PIXImus small animal dual-energy X-ray absorptiometry (DEXA) system (Lunar), as described earlier [11]; BMD of the above genotypes was also measured by peripheral quantitative computerized tomography (pQCT), as described previously [11],[17].

### Skeletal Mineralization

The mineralization pattern of the skeleton was analyzed by Alizarin Red S staining in 6- week-old mice, as described by McLeod [18].

### Histology and Tissue Preparation

All tissues were fixed in 10% buffered formalin. Soft tissues were routinely processed and embedded in paraffin, cut into 4 µm-thick sections and stained with hematoxylin and eosin, and von Kossa.

### Bone Histology and Histomorphometry

Processing of bone specimens and cancellous bone histomorphometry in the distal femoral metaphysis were performed as described [17],[19]. The area within 0.25 mm from the growth plate was excluded from the measurements.

### Biochemical Analyses

Blood was obtained by cheek-pouch bleeding of 3- and 6-week-old animals. Total serum calcium, serum and urinary phosphorus, and serum and urinary creatinine levels were determined using Stanbio LiquiColor (Arsenazo III), Stanbio LiquiUV, and Stanbio Creatinine kits (Stanbio Laboratory, Boerne, TX), respectively. Serum PTH levels were measured using a two-sided enzyme-linked immunosorbent assay (ELISA) specific for intact mouse PTH (Immunotopics, San Clemente, CA, USA). Serum concentrations of 1,25(OH)_2_D_3_ were measured using a radioreceptor assay (Immundiagnostik, Bensheim, Germany). Renal tubular reabsorption of phosphorus (TRP) was calculated according to the formula: %TRP = [1−(UrP×SeCrea)/(SeP×UrCrea)]×100 (Ur, urinary; Se, serum; P, phosphorus; Crea, creatinine).

### Parathyroid Hormone (PTH) Injections

Wild-type, *Fgf-23^−/−^*, and *NaPi2a^−/−^* mice at 4-weeks of age, were injected subcutaneously with vehicle (saline), PTH peptide (1–34), or PTH peptide (3–34) (50 nmol of peptide per Kg of body weight). Blood was collected by cheek-pouch bleeding prior to injections, as well as 2 hours post-injections, and serum phosphate levels were measured using Stanbio LiquiUV kit (Stanbio Laboratory, Boerne, TX).

### *In Situ* Hybridization

Complementary ^35^S-UTP-labeled riboprobes (complementary RNAs for collagen type X (Col X), dentin matrix protein-1 (DMP-1), and osteopontin (OPN)) were used for performing *in situ* hybridization on paraffin sections, as described previously [20].

### Western Blotting

Fresh kidney cortex was isolated from 3 week old mice, and homogenized in HbA buffer (pH 7.4) containing 20 mM Tris base, 5 mM MgCl_2_, 5 mM Na_2_HPO_4_, 1 mM EDTA and 80 mM sucrose and protease inhibitor cocktail tablets (Complete Mini, EDTA-free; Roche). Protein concentration was determined by performing BCA protein assay (Pierce), using BSA as a standard. Protein samples were heated at 95°C for 5 min in sample buffer containing 2% SDS and 1% 2-mercaptoethanol, and were subjected to 10% SDS-polyacrylamide gel electrophoresis. The separated proteins in the gel were transferred electrophoretically to Hybond-P polyvinylidenedifluoride transfer membranes. After incubation in blocking solution, the membranes were further treated with diluted rabbit affinity-purified anti-type 2c NaPi co-transporter antibody (1∶500), a generous gift of Dr Ken-ichi Miyamoto, Japan. Mouse anti-actin monoclonal antibody (SIGMA) was used as an internal control. Horseradish peroxidase-conjugated anti-rabbit or anti-mouse IgG was utilised as the secondary antibody (Jackson ImmunoResearch Laboratories), and signals were detected by the SuperSignal West Pico Chemiluminescent Substrate system (Pierce).

### Mouse Calvarial Cell Culture

Mouse calvarial cell culture was carried out as previously described [21] with modifications. Briefly, mouse calvarial cells were isolated from 3–5 day old C57BL/6J wild-type mice. Calvariae (parietal bones) were removed aseptically, and they were sequentially digested with 2 mg/ml collagenase solution containing collagenase type I and type II in 1∶3 ratio. (collagenase type I and type II; Worthington, Newark, NJ). Osteoblast enriched fractions (the last four of six fractions) were cultured for five to seven days until confluence in α-MEM supplemented with 10% FBS and 1% Penicillin-Streptomycin (Invitrogen Life Technologies, Baltimore, MD). Adherent cells were trypsinized and re-plated at a density of 2.5×10^4^/cm^2^ in the same medium supplemented additionally with 50 µg/ml ascorbic acid and 10 mM β-glycerophosphate (βGP) to induce matrix mineralization with or without treatment with 10 ng/ml of human FGF-23 (hFGF-23). Alizarin Red S staining was performed 21 days after subculture in mineralization medium with or without FGF-23 treatment.

### Statistics

Statistically significant differences between groups were evaluated by Student's t-test for comparison between two groups or by one-way analysis of variance (ANOVA) followed by Tukey's test for multiple comparisons. All values were expressed as mean ±SE. A *p* value of <0.05 was considered to be statistically significant. All analyses were performed using Microsoft Excel and GraphPad Prism 4.0.

## Results

### Generation of *Fgf-23^−/−^/NaPi2a^−/−^* Compound Mutants

*In vivo* ablation of *Fgf-23* results in significantly elevated serum phosphate levels accompanied by enhanced renal phosphate reabsorption and a significant increase in expression and activity of NaPi2a [11],[22],[23]. To test the hypothesis that increased NaPi2a activity is responsible for the severe hyperphosphatemia in *Fgf-23^−^* ^/*−*^ animals, we generated a new mouse model deficient in both the *Fgf-23* and the *NaPi2a* genes (*Fgf-23^−/−^/NaPi2a^−/−^* compound mutants) by interbreeding heterozygous- *Fgf-23* and *NaPi2a* mice. The mice studied were of C57BL/6J genetic background and animals examined were littermates. Compound mutants were viable and were born at the expected Mendelian frequency. In the current study, we compared and analyzed gross phenotypes, and obtained morphological and biochemical data from wild-type, *Fgf-23^−/−^*, *Fgf-23^−/−^/NaPi2a^−/−^*, and *NaPi2a^−/−^* animals.

### Macroscopic Phenotype of *Fgf-23^−/−^/NaPi2a^−/−^* Compound Mutants

**Figure 1:**
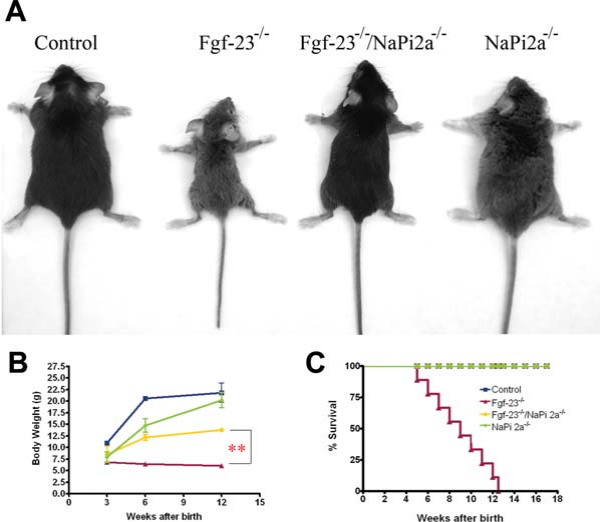
Macroscopic phenotype of *Fgf-23* *^−^* ^*/−*^ */NaPi2a* *^−^* ^*/−*^ double mutants. (A) Gross phenotype of control, *Fgf-23^−/−^*, *Fgf-23^−/−^/NaPi2a^−/−^*, and *NaPi2a^−/−^* animals at 6 weeks of age. (B) Growth curves and (C) survival ratios for all four genotypes. Data are represented as mean ±SEM (** p<0.01).

At birth, *Fgf-23^−/−^/NaPi2a^−/−^* mice appear indistinguishable from their normal littermates. At 3 weeks *Fgf-23^−/−^/NaPi2a^−/−^* compound mutants are larger in size than *Fgf-23^−/−^* mice (8.6±1.7 g *vs* 6.8±0.38 g), but are slightly smaller than wild-type (10.9±0.2 g), and similar to *NaPi2a^−/−^* single knock-out animals (7.9±1.1 g). At 6 and 12 weeks of age, compound mutants are still smaller than wild-type littermates (12.2±0.7 g *vs* 20.7±0.2 g at 6 weeks), but their body weight is significantly higher than that of *Fgf-23^−/−^* mice (6.5±0.2 g) (Figure 1A and B). Apart from the slightly reduced body size, double mutants do not show any obvious gross abnormalities with regard to movement and physical activities, whereas *Fgf-23^−/−^* littermates have severely weakened and restricted movement, as well as sluggish physical activities. In addition, *Fgf-23^−/−^/NaPi2a^−/−^* survive longer than *Fgf-23^−/−^* mice (Figure 1C).

### Bone Densitometry

**Figure 2:**
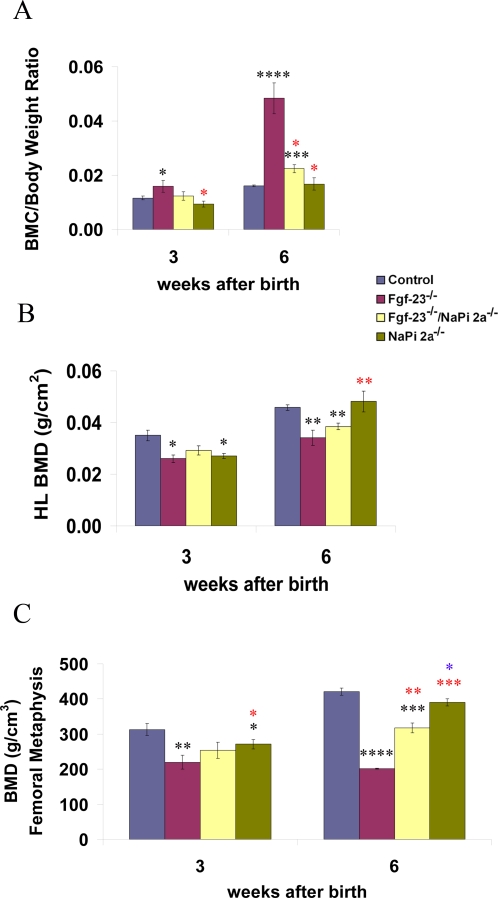
Bone mineralization analysis. (A) Total bone mineral content (BMC; each value obtained for BMC was normalized to the body weight of the corresponding animal). (B) Bone mineral density (BMD) of hind-limbs by Piximus, and (C) pQCT of control, *Fgf-23^−/−^*, *Fgf-23^−/−^/NaPi2a^−/−^*, and *NaPi2a^−/−^* animals. (Statistical significance * p<0.05, ** p<0.01,*** p<0.001. Black asterisks represent comparison with control, red with *Fgf-23^−/−^,* and blue with *Fgf-23^−/−^/NaPi2a^−/−^*).

To evaluate the effects of *NaPi2a* gene ablation on the skeleton of *Fgf-23^−/−^* animals, bone densitometric measurements of hind limbs from 3- and 6-week old control, *Fgf-23^−/−^*, *Fgf-23^−/−^/NapI2a^−/−^*, and *NaPi2a^−/−^* littermates were carried out using PIXImus and pQCT (Figure 2). PIXImus analysis showed significantly increased total body bone mineral content (BMC) in *Fgf-23^−/−^* mice when compared to wild-type controls at both ages (0.016±0.002 *vs*. 0.012±0.0007 at 3 weeks and 0.048±0.006 *vs*. 0.016±0.0003 at 6 weeks) (Figure 2A). In contrast, the BMC of *Fgf-23^−/−^/NaPi2a^−/−^* compound mutants was similar to control littermates at 3 weeks (0.012±0.002) but it was significantly elevated at 6 weeks (0.0225±0.001), although it was significantly lower when compared to *Fgf-23^−/−^* mice (Figure 2A). In accordance with previous reports [9], the total body BMC of *NaPi2a^−/−^* mice at both ages was comparable to wild-type animals (Figure 2A).

Bone densitometric measurements using PIXImus and pQCT demonstrated decreased areal and volumetric bone mineral density (BMD) in the hindlimbs and in the distal femoral metaphysis of *Fgf-23^−/−^* mutant mice at both 3 and 6 weeks of age (Figure 2B and C). The bones of *NaPi2a^−/−^* single knock-out mice demonstrated a significantly reduced BMD at 3 weeks, which was nearly normalized by 6 weeks (Figure 2B and C), in accord with earlier published observations [9]. Areal and volumetric BMD of *Fgf-23^−/−^/NaPi2a^−/−^* compound mutants was not significantly different from that of control littermates at 3 weeks (Figure 2B and C). However, at 6 weeks, volumetric BMD was significantly higher in compound mutants compared with *Fgf-23^−/−^* mice, but still lower than in wild-type controls (Figure 2C).

### Serum and Urine Biochemical Parameters

**Figure 3:**
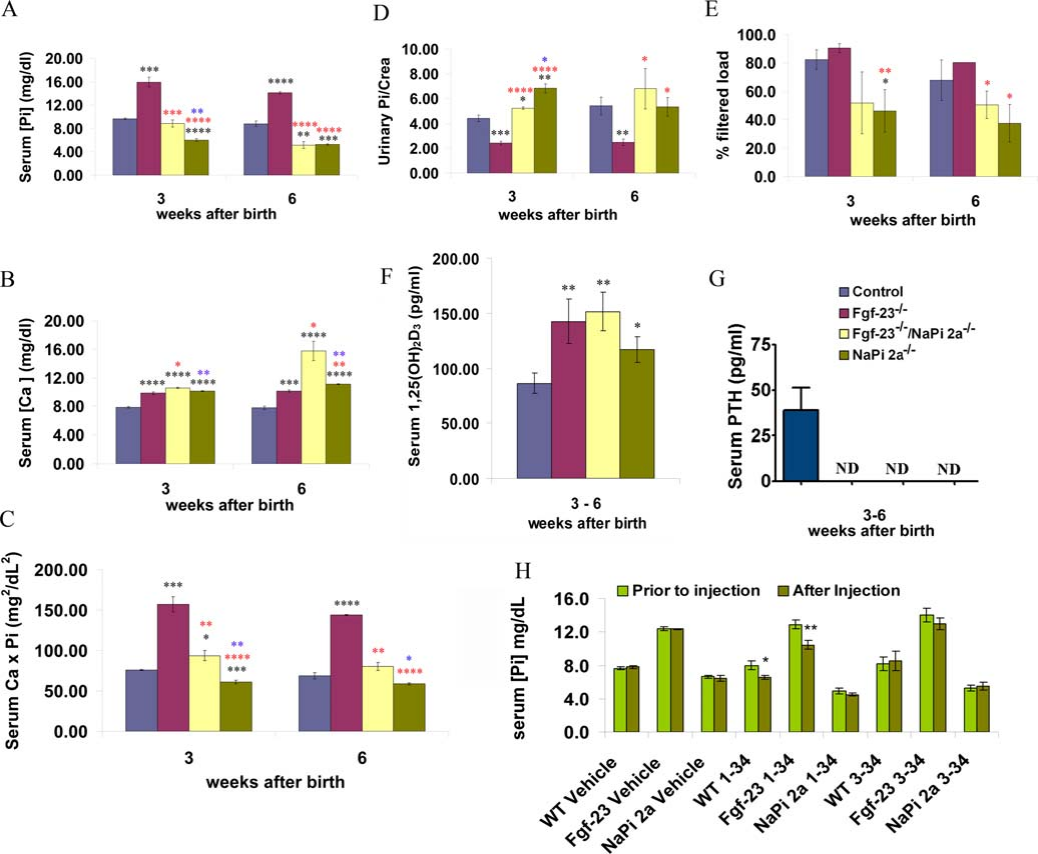
Biochemical measurements. (A) serum phosphate, (B) serum calcium, (C) calcium-phosphate product, (D) urinary phosphate, (E) fractional renal tubular reabsorption of phosphate (TRP), (F) serum 1,25(OH)_2_D_3_, and (G) serum PTH levels in control, *Fgf-23^−/−^*, *Fgf-23^−/−^/NaPi2a^−/−^*, and *NaPi2a^−/−^* animals. (H) serum phosphate levels before and after injections with vehicle, PTH (1–34) or PTH (3–34). (Statistical significance *p<0.05, **p<0.01, *** p<0.001. Black asterisks represent comparison with control, red with *Fgf-23^−/−^,* and blue with *Fgf-23^−/−^/NaPi2a^−/−^*.)

Phosphate, calcium, 1,25(OH)_2_D_3_ and parathyroid hormone (PTH) levels were measured in 3- and 6-week-old wild-type, *Fgf-23^−/−^*, *Fgf-23^−/−^/NaPi2a^−/−^*, and *NaPi2a^−/−^* animals. *Fgf-23^−/−^* mice were severely hyperphosphatemic at both 3 and 6 weeks of age (15.9±0.8 and 14.1±0.2 mg/dl, respectively) when compared to control littermates (9.6±0.1 and 8.8±0.4 mg/dl, respectively). However, Fgf-*23^−/−^/NaPi2a^−/−^* animals were normophosphatemic at 3 weeks (8.8±0.6 mg/dl), and became hypophosphatemic with significantly lower serum phosphate levels (5.2±0.6 mg/dl) by 6 weeks, comparable to those found in *NaPi2a^−/−^* animals of the same age (5.3±0.1 mg/dl) (Figure 3A). More importantly, decreased urinary phosphate excretion (normalized to urinary creatinine) in *Fgf-23^−/−^* mice (2.4±0.1 *vs* 4.4±0.2 in control littermates at 3 weeks and 2.5±0.3 *vs* 5.4±0.7 at 6 weeks) was reversed in *Fgf-23^−/−^/NapI2a^−/−^* double mutant animals. Compound mutants showed hyperphosphaturia (5.2±0.1 at 3 weeks and 6.8±1.6 at 6 weeks), similar to the one found in *NaPi2a^−/−^* mice (6.8±0.3 and 5.3±0.7 respectively) (Figure 3D). In addition, Fgf-*23^−/−^/NaPi2a^−/−^* animals had reduced fractional renal tubular reabsorption of phosphate (TRP) (51.9±12.4 and 50.4±9.6 % at 3 and 6 weeks respectively) when compared to *Fgf-23^−/−^* mice (90.5±3.2 and 80.4±0.1 %) and wild-type littermates (82.4±6.8 and 67.9±14.3 %) (Figure 3E). Collectively, these results suggest that increased renal phosphate reabsorption due to increased NaPi2a activity is the major cause for abnormal hyperphosphatemia in *Fgf-23^−/−^* mice.

Serum calcium levels were found to be higher in all three mutant mouse lines at 3 weeks of age. At 6 weeks, the calcium levels in *NaPi2a^−/−^* (11.1±0.05 mg/dl) and *Fgf-23^−/−^/NapI2a^−/−^* compound mutants (15.7±1.3 mg/dl) were significantly higher than in *Fgf-23^−/−^* mice (10.1±0.2 mg/dl) (Figure 3B). The considerable elevation in serum calcium levels in all mutants is probably due to excessive vitamin D signaling, as reflected by the significantly increased serum 1,25(OH)_2_D_3_ levels in these mice (Figure 3F). Probably as a result of high serum 1,25(OH)_2_D_3_ and concomitant hypercalcemia, serum PTH was undetectable in all three mutant lines (Figure 3G). The calcium-phosphate product was severely increased in 3- and 6-week-old *Fgf-23^−/−^* mice relative to wild-type controls (Figure 3C). Compound mutants showed only a slight increase in the calcium-phosphate product at 3 weeks, but did not exhibit any significant difference from wild type animals at 6 weeks of age, whereas the calcium-phosphate product was significantly reduced in *Napi2a^−/−^* mice (Figure 3C).

Injection of bioactive PTH peptide (1–34) significantly lowered serum phosphate levels in wild-type and *Fgf-23^−/−^* treated mice, but did not reduce the serum phosphate concentration in *NaPi 2a^−/−^* mice (Figure 3F). Injection of vehicle (saline) or inactive PTH peptide (3–34) had no effect on serum phosphate levels.

### Skeletal Phenotype

**Figure 4:**
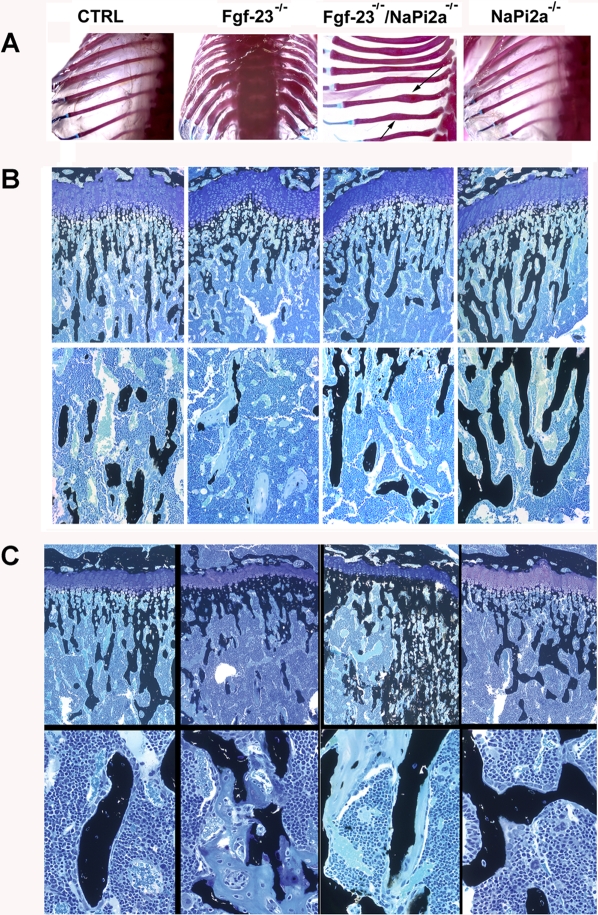
Histological analysis of bones by von Kossa and Alizarin Red S staining. (A) Alizarin Red S stained ribs from all genotypes at 6 weeks of age. (B) Three-µm-thick undecalcified sections from 3- and (C) 6 week-old control, *Fgf-23^−/−^*, *Fgf-23^−/−^/NaPi2a^−/−^,* and *NaPi2a^−/−^* bones were stained with von Kossa/McNeal. *Top panels*: tibial growth plate and trabecular bone (magnification x100); *lower panels*: tibial secondary spongiosa (magnification x400). Black staining represents mineralization. At 6 weeks, more mineral deposition is found in the area below the growth plate (methaphysis) in *Fgf-23^−/−^* mice and *Fgf-23^−/−^/NaPi2a^−/−^* double mutants. In addition, areas of unmineralized osteoid (light blue) are found similarly in the secondary spongiosa of *Fgf-23^−/−^* and *Fgf-23^−/−^/NaPi2a^−/−^* mice.

To examine the mineralization pattern of the bones, Alizarin Red S staining was performed on full body skeletons of 6-week-old *Fgf-23^−/−^/NaPi2a^−/−^* mutants and was compared to wild-type, *Fgf-23^−/−^*, and *NaPi2a^−/−^* animals. The skeletal phenotype of *Fgf-23^−/−^/NaPi2a^−/−^* compound mutants resembled the one seen in *Fgf-23^−/−^* animals with typically widened ribs, whereas bones from *NaPi2a^−/−^* mutant mice were comparable to wild-type mice (Figure 4A).

In agreement with the bone densitometric data, histological analysis of methylmethacrylate sections from femurs showed almost normal bone architecture in *Fgf-23^−/−^/NaPi2a^−/−^* double mutants at 3 weeks (Figure 4B). In contrast, the histological bone phenotype of *Fgf-23^−/−^/NaPi2a^−/−^* double mutants closely resembled that of *Fgf-23^−/−^* mice at 6 weeks (Figure 4C). The bones of 6-week old *Fgf-23^−/−^* and *Fgf-23^−/−^/NaPi2a^−/−^* mice exhibited a decreased number of hypertrophic chondrocytes, hypermineralization adjacent to the growth plate in the primary spongiosa, and severe osteoidosis in the secondary spongiosa (Figure 4B). Bones from *NaPi2a^−/−^* mice at 3 and 6 weeks appeared normal at the histological level (Figure 4B).

Quantitative histomorphometry (Table 1) revealed a striking increase in osteoid volume and osteoid thickness in *Fgf-23^−/−^* mice at 3 and 6 weeks of age. Interestingly, osteoid thickness was normal in 3-week-old compound mutants and *NaPi2a^−/−^* animals, although osteoid volume and surface was increased in *NaPi2a^−/−^* mice relative to wild-type controls. Similar to the histological appearance of the bones, histomorphometry confirmed the severe mineralization defect in *Fgf-23^−/−^* mice and *Fgf-23^−/−^/NaPi2a^−/−^* compound mutants at 6 weeks of age, as evidenced by similar increases in osteoid volume and thickness relative to wild-type mice. Six-week-old *NaPi2a^−/−^* mice had normal osteoid thickness and osteoid volume.

**Table 1:** Bone histomorphometry data.

Variable	Control	*Fgf-23^−/−^*	*Fgf-23^−/−^/NaPi2a^−/−^*	*NaPi2a^−/−^*
**3 weeks**				
BV/TV (%)	13.74±6.39	13.75±8.95	8.98±4.62	10.44±4.36
OV/BV (%)	7.36±2.31	32.09±17.42 ^a^*	12.16±7.03 ^a†^	19.24±8.19 ^a^*
OS/BS (%)	25.66±4.32	52.48±6.66 ^b^*	38.48±7.34	42.80±7.49 ^a^*
ObS/BS (%)	12.61±6.98	7.48±5.25	12.70±7.83	20.39±10.95
OTh (µm)	2.83±0.58	6.92±1.79 ^b^*	3.11±0.73^ b†^	3.48±0.74 ^b†^
TbTh (µm)	16.05±2.45	17.55±1.61	16.29±3.01	16.00±4.79
TbSp (µm)	116.94±48.97	159.5±115.78	226.32±151.89	169.35±120.82
TbN (1/mm)	8.56±3.56	7.60±4.30	5.54±3.11	7.61±4.95
**6 weeks**				
BV/TV (%)	9.88±3.70	10.56±3.02	26.29±8.43	14.63±11.92
OV/BV (%)	3.04±2.18	47.44±11.67 ^d^*	27.44±8.34 ^d^*	5.78±2.67 ^a‡, b†^
OS/BS (%)	14.28±4.28	42.30±3.40 ^b^*	42.89±7.99 ^b^*	30.79±8.86
ObS/BS (%)	16.75±6.76	5.26±5.04	4.19±0.60	19.51±4.56
OTh (µm)	2.39±0.95	10.11±3.84 ^c^*	11.16±2.85 ^d^*	2.98±0.94 ^a†, b‡^
TbTh (µm)	24.81±4.49	25.20±1.16	37.06±9.94	28.76±9.36
TbSp (µm)	242.83±57.39	230.04±87.74	111.94±54.84	218.98±104.24
TbN (1/mm)	3.89±0.91	4.20±1.22	7.36±2.50	4.61±2.24

BV/TV, bone volume; OV/BV, osteoid volume; OS/BS, osteoid surface; ObS/BS, osteoblast surface; OTh osteoid thickness; TbTh, trabecular thickness; TbSp, trabecular separation; TbN, trabecular number. ^a^ p<0.05, ^b^ p<0.01, ^c^ p<0.001. * represents comparison with control, † with *Fgf-23^−/−^,* and ‡ with *Fgf-23^−/−^/NaPi2a^−/−^*.

Collectively these data demonstrate that the defect in bone mineralization seen in hyperphosphatemic *Fgf-23* mutants is also present in 6-week-old hypophosphatemic *Fgf-23^−/−^/NaPi2a^−/−^* mice despite the opposite serum phosphate levels. Thus, the mineralization defect in Fgf-23*^−^* ^/*−*^ mutants and *Fgf-23^−/−^/NaPi2a^−/−^* compound mutants appears to be due to lack of Fgf-23 gene expression rather than systemic phosphate homeostasis. Moreover, NaPi2a*^−^* ^/*−*^ littermates which completely resemble the serum biochemistry of *Fgf-23^−/−^/NaPi2a^−/−^* animals, did not exhibit any defects in bone mineralization at 6 weeks of age.

### Gene Expression

**Figure 5:**
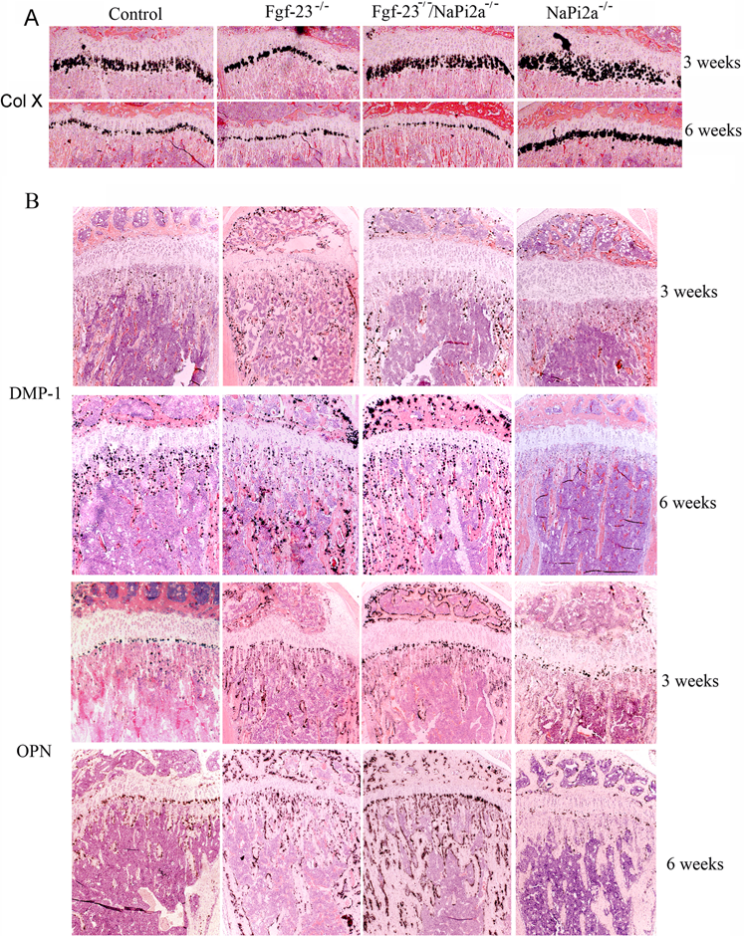
*In situ* hybridization. Riboprobes for (A) collagen type X (Col X), (B) dentin matrix protein-1 (DMP-1), and osteopontin (OPN) on sections from tibia of control, *Fgf-23^−/−^*, *Fgf-23^−/−^/NaPi2a^−/−^,* and *NaPi2a^−/−^*at 3 and 6 weeks.

To analyze the gene expression pattern of bone cells and to examine the effect of *Fgf-23* and *NaPi2a* gene deletion on skeletogenesis, we performed *in situ* hybridization on paraffin sections prepared from tibias of wild-type, *Fgf-23^−/−^*, *Fgf-23^−/−^/NaPi2a^−/−^*, and *NaPi2a^−/−^* animals at 3 and 6 weeks of age (Figure 5). Similar to our previous findings [11],[23], the number of hypertrophic chondrocytes was reduced in *Fgf-23^−/−^* animals at both ages, relative to control mice, as demonstrated by the marked decrease in collagen type X expression (Figure 5A). Similarly, *Fgf-23^−/−^/NaPi2a^−/−^* compound mutants also showed a significant reduction in the number of hypertrophic chondrocytes at 6 weeks, comparable to *Fgf-23^−/−^* animals, although collagen type X expression at 3 weeks was normal in these mice (Figure 5A). In contrast, we noted a marked increase of collagen type X-positive cells in *NaPi2a^−/−^* mice, especially at 3 weeks of age (Figure 5A). Furthermore, we examined expression of osteopontin (OPN) and dentin matrix protein (DMP-1), two members of the SIBLING protein family that exert key biological effects in skeletal mineralization. An association between FGF23 and DMP-1 has been suggested in earlier studies [24]. For instance, increased serum FGF-23 levels were found in patients with autosomal recessive hypophosphatemic rickets (ARHR), a disease caused by mutation in DMP-1 gene [24]. Similarly, in *Dmp-1* null mice Fgf-23 levels were high [25]. In our study, lack of Fgf-23 resulted in increased expression of DMP-1 and OPN in *Fgf-23^−/−^* and *Fgf-23^−/−^/NaPi2a^−/−^* compound mutants at both 3 and 6 weeks. In contrast, *NaPi2a^−/−^* animals exhibited normal DMP-1 and OPN at 3 weeks, however, the expression of both of these genes appeared to be decreased in *NaPi2a^−/−^* animals at 6 weeks, compared to wild-type controls (Figure 5B).

### Morphology of Soft Tissue Anomalies in *Fgf-23^−/−^/NaPi2a^−/−^* Mice

**Figure 6:**
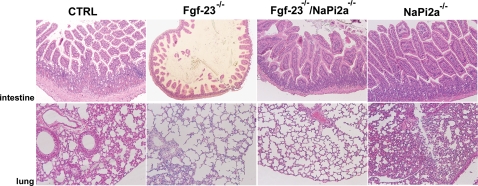
Histological analysis of soft tissues. Hematoxylin and Eosin-stained sections of intestines and lungs from 6 week-old control, *Fgf-23^−/−^*, *Fgf-23^−/−^/NaPi2a^−/−^,* and *NaPi2a^−/−^*. Intestinal sections from *Fgf-23^−/−^* mice reveal reduced height of intestinal villi and atrophy of intestinal mucosa. In addition, *Fgf-23^−/−^* mice exhibit lung emphysema. These features are significantly improved in *Fgf-23^−/−^/NaPi2a^−/−^* mice (magnification ×2.5).

Histological examination of various soft tissues from *Fgf-23^−/−^/ NaPi2a^−/−^* double mutants showed that abnormalities such as intestinal atrophy and lung emphysema that are consistently observed in single *Fgf-23^−/−^* animals, were ameliorated, but not completely abolished in *Fgf-23^−/−^/NaPi2a^−/−^* double mutants, suggesting that these soft tissue pathological changes are partially caused by the severely increased serum phosphate levels in *Fgf-23^−/−^* mice (Figure 6).

### Effect of FGF-23 on the Mineralization of Mouse Calvarial Osteoblasts

**Figure 7:**
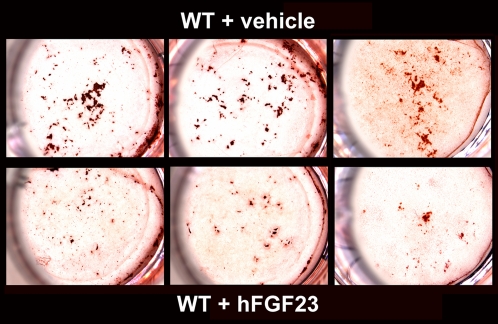
Alizarin Red S staining of wild-type calvarial osteoblasts treated with either mineralization medium alone or with medium containing hFGF23 protein for 21 days. Top panels show vehicle treated wild-type cells (n = 15) and bottom panels show wild-type cells treated with hFGF-23 (n = 15).

To evaluate the effect of FGF-23 on mineralization we cultured osteoblastic cells isolated from C57BL/6J wild-type calvariae in mineralization medium alone (vehicle) or mineralization medium containing hFGF-23 protein. Alizarin Red S staining was carried out after 21 days. A marked decrease in mineralized bone nodule formation was evident in cells treated with hFGF-23 when compared to vehicle treated cells (Figure 7). These data suggest that excess of FGF-23 in osteoblast cultures leads to an impairment of mineralization *in vitro*.

## Discussion

This is the first study using a genetic mouse model with dual ablation of the *NaPi2a* and *Fgf-23* genes. *Fgf-23^−/−^* mice develop severe hypercalcemia, hyperphosphatemia, hypervitaminosis D, and osteomalacia starting in early life [11],[22]. The hyperphosphatemia in *Fgf-23^−/−^* mice is associated with increased renal phosphate uptake, and increased expression of the renal Na/Pi2a co-transporter in the proximal tubular epithelial cells [26]. In our study, ablation of both *NaPi2a* co-transporter and *Fgf-23* in the same animal resulted in reduced serum phosphate levels which were accompanied by increased urinary phosphate excretion in *Fgf-23^−/−^/NaPi2a^−/−^* mice, reemphasizing the fact that increased NaPi2a activity in the renal proximal tubular epithelial cells is responsible for the severe hyperphosphatemia in *Fgf-23^−/−^* mice. These results provide compelling genetic evidence of the importance of NaPi2a in regulating renal phosphate homeostasis in *Fgf-23^−/−^* mice. Deletion of *NaPi2a* from these animals and accompanied changes in serum phosphate levels significantly improved the abnormal phenotype associated with lack of Fgf-23 activities, indicating that the high phosphate microenvironment contributes to the development of widespread soft tissue atrophy and calcifications in *Fgf-23^−/−^* mice. Similar observations were made in recent studies in which the increased vitamin D signaling in *Fgf-23^−/−^* mice was blocked by additionally ablating the renal 1α-hydroxylase or the vitamin D receptor [23],[27], or in which *Fgf-23^−/−^* mice were fed a low phosphate diet [3]. In addition, our study suggests that hypervitaminosis D is toxic when associated with an increased calcium-phosphate product.

The mechanisms behind the upregulation of NaPi2a expression and activity in *Fgf-23^−/−^* mice are still poorly understood. The retrieval and recruitment of NaPi2a proteins is a complex multi-factorial process, and the *in vivo* interactions between FGF-23, vitamin D, PTH, and NaPi2a transporters need additional studies for comprehensive understanding. Earlier studies have shown that administration of FGF-23 increases urinary phosphate excretion by suppressing renal expression of sodium-phosphate co-transporters [28]. Therefore, the upregulation in NaPi2a protein in *Fgf-23^−/−^* mice may be a direct effect of *Fgf-23* ablation. On the other hand, high 1,25(OH)_2_D_3_ and suppressed PTH in *Fgf-23^−/−^* mice could also be involved. PTH is a powerful inhibitor of renal phosphate reabsorption by facilitating endocytosis of the NaPi2a transporters from the brush-border membrane of proximal tubular epithelial cells for eventual lysosomal degradation [29],[30]. To test the hypothesis that suppressed serum PTH in *Fgf-23^−/−^* mice could diminish or delay the endocytosis of the NaPi2a transporters from the proximal tubular epithelial cells, we injected vehicle (saline), PTH (1–34) and PTH (3–34) into *Fgf-23^−/−^* and *NaPi2a^−/−^* mice. We found that a single injection of bioactive PTH (1–34) can significantly reduce serum phosphate levels in wild-type and *Fgf-23^−/−^* mice (Figure 3H). In contrast, no effect of PTH (1–34) injection on serum phosphate levels was noted in *NaPi2a^−/−^* mice emphasizing that Napi2a is the dominant sodium phosphate co-transporter in the renal proximal tubule cells and responsible for the severe hyperphosphatemia in *Fgf-23^−/−^* mice. As expected, injections of vehicle or inactive PTH (3–34) did not have any effect on serum phosphate levels in all mice examined. NaPi2c, another sodium phosphate co-transporter in the renal proximal tubule cells was upregulated in *Fgf-23^−/−^*, *Fgf-23^−/−^*/*NaPi2a^−/−^*, and *NaPi2a^−/−^* mice when compared to wild type littermates (Figure S1). From these results we conclude that 1) reduced level of PTH in *Fgf-23^−/−^* mice could contribute to the upregulation of NaPi2a expression in these mice and thereby to the development of hyperphosphatemia, and 2) compensatory increased expression of NaPi2c cannot efficiently restore the effects of NaPi2a loss.

The main source of Fgf-23 production has been shown to be the osteocyte [23],[31]. We, therefore, analyzed the skeleton of *Fgf-23^−/−^/NaPi2a^−/−^* compound mutants, in which serum phosphate levels were reversed to hypophosphatemia. Surprisingly, skeletal abnormalities observed in *Fgf-23^−/−^* mice including the decrease in hypertrophic chondrocytes in the growth plate, the increased mineral deposition adjacent to the growth plate, and the osteomalacic phenotype were found to be similar in 6-week-old *Fgf-23^−/−^/NaPi2a^−/−^* compound mutants, despite their significantly reduced serum phosphate levels. Furthermore, our data conclusively show that the osteomalacic phenotype in *Fgf-23^−/−^* and *Fgf-23^−/−^/NaPi2a^−/−^* compound mutants at 6 weeks of age is not caused by changes in serum phosphate levels. Rather, our findings suggest that the increased 1,25(OH)_2_D_3_ serum levels, possibly in combination with elevated serum calcium-phosphate levels, cause osteomalacia in *Fgf-23^−/−^* mice. In line with this notion, studies have convincingly demonstrated that rats treated with high doses of 1,25(OH)_2_D_3_ have impaired bone mineralization [32],[33].

A recent study has demonstrated that NaPi2a is expressed in mouse MC3T3-E1 and rat UMR-106 osteoblast-like cells and its expression is regulated by phosphate [34], supporting a role of NaPi2a in mediating phosphate transport in osteoblasts. Therefore ablation of NaPi2a could affect bone mineralization. However, although both *NaPi2a^−/−^* and *Fgf-23^−/−^/NaPi2a^−/−^* compound mutants lack NaPi2a and have similar biochemical parameters, they exhibit a different skeletal phenotype. One obvious difference between these two mouse models however is the lack of Fgf-23 expression, suggesting that Fgf-23 has a significant role in bone mineralization. This hypothesis is strengthened by *in vitro* studies by Wang *et al* in which they show that adenoviral overexpression of FGF-23 in rat calvarial cells inhibits bone mineralization independent of systemic effects on phosphate homeostasis [35]. In addition, we have pursued *ex vivo-in vitro* studies by isolating and culturing mouse calvarial osteoblasts from wild-type mice and exposing them to FGF-23 treatment. Our data demonstrate that FGF-23 treatment of primary calvarial osteoblasts from wild-type mice leads to an inhibition of mineralization as shown by the decrease in Alizarin staining (Figure 7). Moreover, we could confirm the previously published data which show a reduction in mineralization using osteoblasts isolated from *Hyp* mice, which produce high levels of Fgf-23, again emphasizing that FGF-23 is a potent inhibitor of mineralization [36,37 and data not shown]. Taken together, these results suggest that excess of FGF-23 can negatively regulate bone mineralization. However, the mechanism responsible for the effect of FGF-23 on bone mineralization, as well as the role of Klotho, if any, in the Fgf-23-specific signaling in osteoblasts *in vivo* remain to be determined.

The expression pattern of the two sibling proteins, OPN and DMP-1 in bones of wild type, *Fgf-23^−/−^* and *Fgf-23^−/−^/NaPi2a^−/−^* and *NaPi2a^−/−^* at 3 and 6 weeks of age demonstrated phosphate independent effect of Fgf-23 on bone. Previous *in vitro* studies using wild-type murine cementoblasts, have shown phosphate-dependent regulation of DMP-1 and OPN [38]. Interestingly, however, we have found that expression of DMP-1 and OPN in bones from *Fgf-23^−/−^* and *Fgf-23^−/−^/NaPi2a^−/−^* compound mutants is significantly upregulated at 3 and 6 weeks of age. Thus, in the absence of Fgf-23 activity, increased expression of DMP-1 and OPN appears to be independent of circulating phosphate levels and might, therefore, be partly mediated through direct effects of Fgf-23 on these SIBLING genes, but such a hypothesis needs to be further investigated.

In summary, the phenotype of *Fgf-23^−/−^/NaPi2a^−/−^* compound mutants demonstrates that 1) increased NaPi2a activity is the main cause for the severe hyperphosphatemia observed in *Fgf-23^−/−^* mice, 2) that the mineralization defect and the growth plate changes in Fgf-23*^−^* ^/*−*^ and *Fgf-23^−/−^/NaPi2a^−/−^* compound mutants at 6 weeks of age are partly due to lack of Fgf-23 function rather than systemic phosphate homeostasis, and 3) that the altered expression of the sibling proteins OPN and DMP1 in bone is independent of serum phosphate levels in mice ablated for Fgf-23. Genetic ablation of *NaPi2a* from *Fgf-23^−/−^* mice reversed the hyperphosphatemia to hypophosphatemia, and partially improved the soft tissue calcifications and atrophy. Analysis of the bones from *Fgf-23^−/−^/NaPi2a^−/−^* compound mutants revealed that the osteomalacic bone phenotype in mice lacking Fgf-23 is not always associated with serum phosphate levels. Further analyses are needed to determine the detailed molecular interactions of Fgf-23 with genes responsible for skeletal mineralization.

## Supporting Information

Figure S1 Expression of NaPi2c in renal cortex by Western Blotting. Actin was used as internal control.
(1.77 MB TIF)
