# Supplementary figures and images for: Genetic Evidence of Serum Phosphate-Independent Functions of FGF-23 on Bone
Source: PLoS Genet. 2008 Aug 8;4(8):e1000154. doi: 10.1371/journal.pgen.1000154 (PMC2483943; doi:10.1371/journal.pgen.1000154)

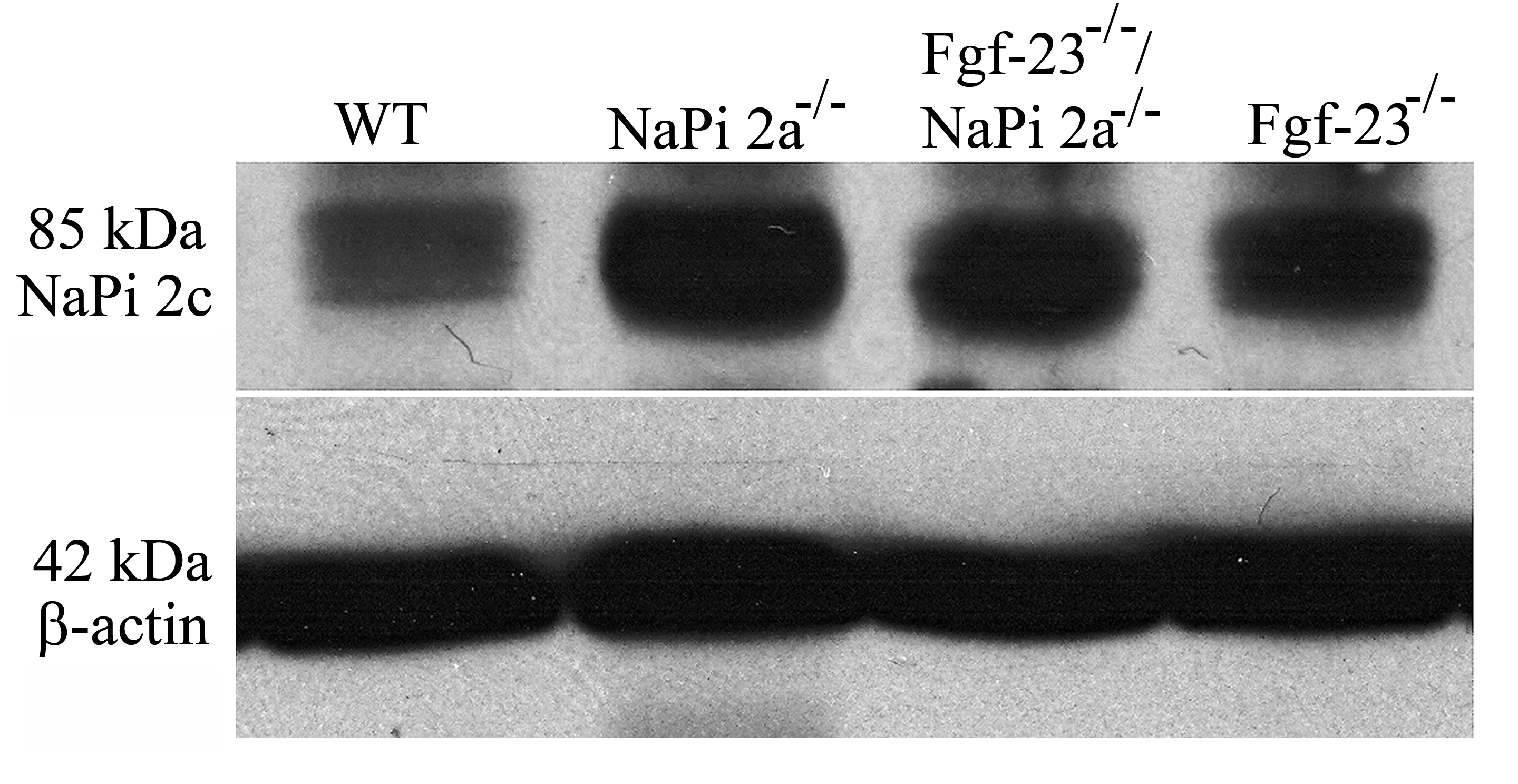

Supplement: Figure S1 — Expression of NaPi2c in renal cortex by Western Blotting. Actin was used as internal control. (1.77 MB TIF) [file pgen.1000154.s001.tif]
